# Comparative Study of Chemical Compositions and Antioxidant Capacities of Oils Obtained from Sixteen Oat Cultivars in China

**DOI:** 10.3390/foods14122007

**Published:** 2025-06-06

**Authors:** Feiyue Ma, Taotao Dai, Laichun Guo, Chunlong Wang, Changhong Li, Chunhua Li, Jun Chen, Changzhong Ren

**Affiliations:** 1 State Key Laboratory of Food Science and Technology, Nanchang University, Nanchang 330047, China; mafeiyuefenglingcao@126.com (F.M.); daitaotao@ncu.edu.com (T.D.);; 2 National Oat Improvement Center, Baicheng Academy of Agricultural Sciences, Baicheng 137000, Chinalichunhua2007@hotmail.com (C.L.)

**Keywords:** oat oil, cultivars, lipid concomitants, antioxidant capacity

## Abstract

The global oat harvest area occupied by China has been increasing annually. In this study, the fatty acid and triacylglycerol compositions, lipid concomitants, and antioxidant capacities of 16 oat oil cultivars in China were compared. All oat oils were found to be rich in unsaturated fatty acids (UFA), particularly oleic acid and linoleic acid. The main triacylglycerols in oat oil were first reported, including 1-palmitoyl-2-linoleoyl-3-oleyl-glycerol (PLO, 16.50–18.69%), 1,3-dioleoyl-2-linoleoyl-glycerol (OLO, 14.97–18.44%), and 1-palmitoyl-2,3-dioleoyl-glycerol (POO, 11.00–13.45%). Significant variations were observed among the cultivars in lipid concomitants, including tocochromanols (0–124.83 mg/kg), phytosterols (3380.94–5735.96 mg/kg), squalene (17.39–59.33 mg/kg), and polyphenols (255.47–513.99 mg GAE/kg). The antioxidant capacities of the different cultivars varied for DPPH (154.34–189.80 μmol VE/kg), ABTS (124.40–343.97 μmol VE/kg), and FRAP (834.32–2746.09 μmol VE/kg). Pearson correlation analysis showed a positive correlation between antioxidant capacity and the contents of polyphenols, squalene, and campesterol. Hierarchical cluster analysis classified the oat oils into distinct groups based on their phytosterol, polyphenol, monounsaturated fatty acids (MUFA), triacylglycerol, squalene, polyunsaturated fatty acid (PUFA), and tocochromanol contents. This study confirms that oat oil has potential as a functional oil and dietary supplement, and sheds light on the relationship between its nutritional quality and functionality, which may aid in the screening of beneficial oat oil cultivars.

## 1. Introduction

Common oat (*Avena nuda* L.), an allohexaploid species (2n = 6x = 42, genomes AACCDD) in the Poaceae family, are a valuable source of minerals, proteins, fibers, lipids, unsaturated fatty acids, vitamins, and phytochemicals [1,2,3]. Traditionally used as high-quality forage, oats are increasingly valued for their applications in the food and pharmaceutical industries, owing to their high protein, dietary fiber, and bioactive compound content, including β-glucans, specific oils, and antioxidants [4,5]. There are significant differences in the chemical composition across different cultivars overseas. This has spurred research into optimizing cultivars with tailored chemical profiles to enhance yield and bioactive compound content [6]. Global oat production has garnered increasing attention in recent years. Based on the data released by FAOSTAT, between 2018 and 2022, the harvested area for oats worldwide ranged from 9.57 to 9.98 million hectares, with the global yield reaching 196,520 kg/ha in 2022. During this period, China’s share of the world’s oat harvest area steadily increased, reflecting a heightened national interest in oat cultivation. Despite this progress, the research on the chemical and bioactive components of oats is still insufficient.

Oat oil is a significant component of oats, comprising roughly 2–18% [5,7]. Oat oil improves the skin barrier and protects effect on reprotoxicity [7]. A distinguishing feature of oat oil is its unique storage mechanism. Unlike most cereals, which accumulate oil primarily in the embryo, scutellum, or aleurone layers, oats deposit up to 90% of their oil within the endosperm—the same cells that store starch [7,8,9]. The composition of oat oil, including polyphenolics, tocochromanols, and phytosterols, varies by cultivar and growing location [10,11] These components contribute to a wide range of pharmacological applications, antioxidants, antimutagenic, anti-inflammatory and particularly in combating chronic diseases such as cardiovascular conditions [5,12]. Despite growing interest, the chemical composition and antioxidant capacity of oat oils cultivated in China remain poorly characterized, hindering the selection of optimal cultivars for targeted applications based on active compounds or functional properties.

In this study, we compared the composition of 16 oat cultivars grown in China, analyzing their fatty acid profiles, triacylglycerol content, lipid concomitants, and antioxidant capacities to assess their potential in the food, pharmaceutical, and cosmetic industries. Pearson correlation analysis was conducted to evaluate the relationships between major lipid concomitants and antioxidant capacities, while hierarchical cluster analysis (HCA) was used to assess the similarity among the 16 cultivars. These findings provide essential data for evaluating the quality and nutritional value of commercial oat oil, offering insights into oat breeding and potential industrial applications.

## 2. Materials and Methods

### 2.1. Materials and Chemicals

Sixteen oat seed cultivars (1# = BaiYan 1#, 2# = BaiYan 2#, 3# = BaiYan 3#, 4# = BaiYan 4#, 5# = BaiYan 5#, 8# = BaiYan 8#, 9# = BaiYan 9#, 10# = BaiYan 10#, 11# = BaiYan 11#, 13# = BaiYan 13#, 15# = BaiYan 15#, 16# = BaiYan 16#, 18# = BaiYan 18#, 20# = BaiYan 20#, 22# = BaiYan 22#, 24# = BaiYan 24#) were collected from National Oat Improvement Center, which was located in Baicheng City (latitude: 45°26′28.76″, longitude: 122°49′13.44″), Jilin Province, China in August 2023. The growing conditions and agronomic management protocols were as follows: annual mean temperature: 5.2 °C, annual mean precipitation: 19.7 mm, fertilization rates: 250 kg/ha (N-P_2_O_5_-K_2_O), and water once each at the three-leaf stage and the filling stage. Further details regarding the cultivars are shown in Table S1.

Standards of Supelco™ 37 Component FAME Mix C_4_-C_24_, triacylglycerol, eight tocochromanols, phytosterols, squalane, and 1, 1-diphenyl-2-pireyhydrazyl (DPPH), and chromatographic grade methanol were purchased from Sigma-Aldrich Co. (Steinheim, Germany). The total Antioxidant Capacity Assay Kit with the FRAP method and the Total Antioxidant Capacity Assay Kit with the ABTS method were purchased from Beyotime Biotech Inc. (Shanghai, China). Other reagents were of analytical grade and purchased from Kermel Chemical Reagents Corp. (Tianjin, China).

### 2.2. Proximate Analysis of Oat Seeds

The moisture, ash, crude protein, lipid and starch content of dried oat seeds were determined by the hot air drying at 120 °C (AOAC Method 990.19), hot air drying at 550 °C (AOAC Method 923.03), Kjeldahl (N × 5.83) (AOAC Method 979.09), Soxhlet extraction (AOAC Method 963.15), and enzymatic digestion (AOAC Method 2002.02), respectively. A colorimeter (CM-5, Hangzhou Ke Sheng Instrument Co. Ltd., Hangzhou, China) was used to evaluate the color variables (L*, a* and b*) of the different oat cultivars.

### 2.3. Oat Oil Extraction Process

Oats were collected and pretreated (cleaned and dried by the sun). The dried oats were milled using a disintegrator (HX-200A, Yongkang Hardware and Medical Instrument Plant, China) and passed through a 40-mesh sieve. Then, the oat oils were extracted from flour according to the method reported by Shuai et al. [2]. The unconsumed oat flours were packed with oxygen insulation and stored at 20 °C for further analysis. In brief, each variety of oat flour (1000 g) and *n*-hexane (6000 mL) were mixed at 50 °C for 6.0 h at 500 r/min. Thereafter, the extract was filtered, and *n*-hexane in the filtrate was removed at 50 °C under reduced pressure by a rotary evaporator (Hei-vap Precision, Heidolph Co., Schwabach, Germany). The obtained oat oil’s approximate volume was about 40–70 mL. The composition and content of oat oils were further analyzed.

### 2.4. Fatty Acid and Triacylglycerol Compositions Analysis

According to the previous research [13], briefly, 0.25 g of oat oil was mixed with 10.0 mL of *n*-hexane. Then, 0.3 mL of KOH-CH_3_OH solution (2.0 mol/L) was added to the mixtures to take the methylation reaction. After methylated, according to the methods of Lakhlifi El Idrissi et al. [14] and Ma et al. [15], the fatty acid composition of oat oils was analyzed by a gas chromatography-flame ionization detector (GC-FID) (Agilent 7890A, Agilent Technologies, Santa Clara, CA, USA) equipped with a BPX capillary column (60 m × 0.22 mm × 0.25 μm). The detailed analytical conditions were as follows: injection volume: 1.0 μL, carrier gas: Nitrogen, flow rate: 1.0 mL/min, heating program: 60 °C (0 min), 60–170 °C (10 °C/min), 170–230 °C (3 °C/min), then the samples were kept for 15 min at 230 °C. The injector and detector temperatures were 225 and 250 °C, respectively. Fatty acid identification was carried out by comparing the retention times with the fatty acid methyl ester standard. The results were presented as percentages of each fatty acid relative to the total (area normalization, %, *w*/*w*).

The triacylglycerol (TAG) composition of oats oil was analyzed using the method with slightly modified from Gao et al. [16] and Shuai et al. [17]. Briefly, 0.05 g of oat oil was dissolved in 5.0 mL of chromatographic-grade *n*-hexanem, and filtered through 0.22 μm organic membrane prior to chromatographic analysis. The GC conditions were as follows: analysis was performed using a gas chromatograph equipped with a DB-17HT capillary column (60 m × 0.22 mm × 0.25 μm) and an FID detector, injection volume: 1.0 μL, nitrogen flow rate: 1.0 mL/min, split ratio: 1:80, injector temperature: 360 °C, detector temperature: 375 °C, heating program: 250 °C (0 min), 250–340 °C (5 °C/min), then kept for 35 min at 340 °C. TAGs of oats oil were identified by comparing the retention times and carbon numbers of TAG standards. The relative percentage of individual TAGs in the total TAGs was calculated using the area normalization method. And the results were expressed as relative proportions.

### 2.5. Lipid Concomitants Analysis

#### 2.5.1. Tocochromanols Analysis

Tocochromanols were qualitatively and quantitatively analyzed using a high-performance liquid chromatographic (HPLC) system (Agilent 1260, Agilent Technologies, Santa Clara, CA, USA) equipped with an ultraviolet (UV) detector and a silica column (4.6 mm × 250 mm, 5 μm, Agilent Technologies, Santa Clara, CA, USA). The detection methods followed the protocol outlined by Shuai et al. [2]. Briefly, a 0.1 g sample was mixed with 10.0 mL of *n*-hexane and then filtered through a 0.45 μm nylon membrane prior to HPLC analysis. The mobile phase consisted of a mixture of n-hexane, methanol, and isopropanol (92.5/7.4/0.1, *v*/*v*/*v*). The injection volume was 20.0 μL, with a flow rate of 1 mL/min and a column temperature set at 30 °C. Tocochromanols were identified and quantified by comparing retention times with standard tocochromanols solutions, and detection was performed at a wavelength of 294 nm.

#### 2.5.2. Phytosterols and Squalene Analysis

Phytosterols and squalene were analyzed using a gas chromatograph-flame ionization detector (GC-FID) system (Agilent Technologies, Santa Clara, CA, USA). For phytosterols, a DB-5MS capillary column (0.25 μm, 30.0 m × 0.25 mm) was used, and for squalene analysis, an HP-5 capillary column (0.25 μm, 30 m × 0.32 mm) was employed. The pretreatment process and detection methods were based on the procedures reported by Shuai et al. [17] and Shuai et al. [18], ensuring high specificity and accuracy in the quantification of these compounds.

#### 2.5.3. The Total Polyphenol Content Analysis

The total polyphenol content in oat oil was determined using the Folin-Ciocalteu method, with modifications based on the protocols by Belhoussaine et al. [19], Gao et al. [20] and Shuai et al. [2]. In this method, 4.0 g of oat oil was mixed thoroughly with 3.0 mL of methanol using a vortex mixer (VX200-T, MET, Rochester, NY, USA) for extraction. The extraction process was conducted in triplicate, and the resulting supernatants obtained from the triplicate extractions were combined and diluted to a final volume of 10 mL. To 0.2 mL of the polar extracts, 0.8 mL of distilled water and 1.0 mL of Folin-Ciocalteu reagent were added. The mixtures were allowed to react for 5 min at room temperature, after which 1.0 mL of 7.5% Na_2_CO_3_ was added. The reaction was then allowed to proceed for 1.5 h in the dark. The absorbance was measured at 760 nm, and the results were expressed as milligrams of gallic acid equivalents per kilogram of oil (mg/kg).

### 2.6. Determination of Antioxidant Capacity

The antioxidant capacity of oat oil was evaluated using three different antioxidant assays: DPPH, ABTS, and FRAP. These methods were employed to assess the free radical-scavenging activities and total antioxidant capacity of the oil, using the methanol extract mentioned in Section 2.5.3. The DPPH assay measures the hydrogen-donating ability or radical-scavenging activity of the samples, as detailed in our previous work [15]. Additionally, the ABTS and FRAP assays were used to assess the total antioxidant capacity. ABTS and FRAP measurements were refined based on previous methods [2,21]. The antioxidant activities of the oat oil samples were expressed as Vitamin E equivalents (VE, μmol/kg), providing a clear measure of the antioxidant potential of each sample.

### 2.7. Statistical Analysis

All samples were conducted in triplicate, and the results were expressed as means ± standard deviations (SD). Statistical analysis was carried out using SPSS 28.0 (SPSS Inc., Chicago, IL, USA). The differences among samples studied were calculated using a one-way analysis of variance (ANOVA) at *p* < 0.05. Hierarchical cluster analysis (HCA) evaluated the similarity among oat oils and the clustering heatmap was drawn using Origin 2023 (OriginLab, Northampton, MA, USA) software packages.

## 3. Results and Discussion

### 3.1. The Appearance and Proximate Composition Analysis of Oat Seed

**Figure 1:**
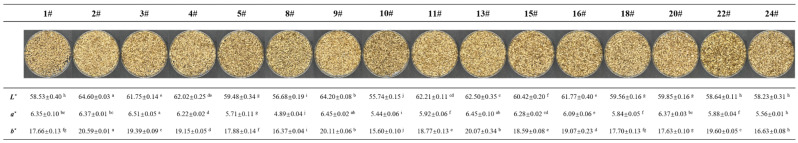
Appearances and colors of sixteen oat cultivars (values were means ± SD. Different letters in a line indicated significant differences (*p* < 0.05)).

Figure 1 presents the shape and color variations among the sixteen oat cultivars. Notable differences in appearance were observed. Most seeds exhibited a spindle shape with smooth surfaces, lacking coleorhiza hair. Among them, cultivars 2# and 20# were visibly larger and fuller. Seed color is an important quality indicator, measured using L*, a*, and b* values, which represent lightness, red-green intensity, and yellow-blue intensity, respectively. Cultivar 2# exhibited the highest L* (64.60) and b* (20.59) values, while cultivar 10# had the lowest L* (55.74), a* (5.44), and b* (15.60) values. Overall, most oat seeds displayed a light-yellow hue, which was reflected in the color of the extracted oil.

**Figure 2:**
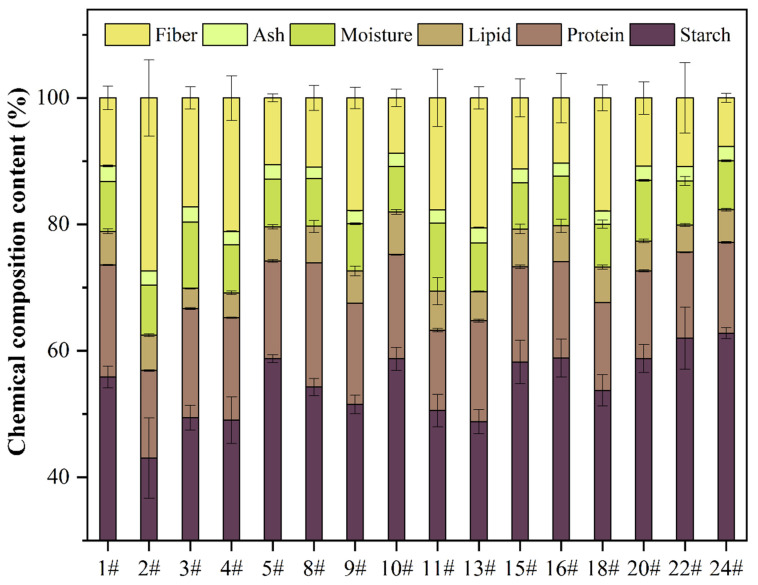
The proximate composition of different cultivars of oat seeds.

Figure 2 illustrates the proximate composition of the different oat cultivars. The moisture content ranged from 6.87 ± 0.66% (cultivar 18#) to 10.80 ± 0.03% (cultivar 11#). Ash content varied from 1.84 ± 0.03% (cultivar 8#) to 2.44 ± 0.12% (cultivar 1#). Protein content ranged from 13.62 ± 0.08% (cultivar 22#) to 19.62 ± 0.04% (cultivar 8#), with cultivar 8# showing a significantly higher protein level than other samples. Starch content ranged from 43.05 ± 6.36% (cultivar 2#) to 62.79 ± 0.88% (cultivar 24#). Lipid content varied between 3.18 ± 0.06% (cultivar 3#) and 6.75 ± 0.41% (cultivar 10#), making oats one of the highest lipid-containing cereal grains. However, unlike maize, where oil is mainly stored in the embryo, oat oil is primarily deposited in the endosperm. Most of the analyzed cultivars fell into the medium-oil category (around 6%). Additionally, the observed protein (12–20%) and starch (50–60%) contents aligned with previous reports [7]. Previous studies suggested a general trend where lipid content increases at the expense of starch [7]. However, our findings did not exhibit a clear correlation.

### 3.2. Fatty Acid and Triacylglycerol Compositions

#### 3.2.1. Fatty Acid Compositions

The composition of fatty acids plays a significant role when considering the quality and nutrition of oil. As shown in Table 1, thirteen major fatty acids were identified in the oat oils analyzed in this study. The predominant fatty acids were palmitic acid (C16:0), oleic acid (C18:1), and linoleic acid (C18:2), collectively accounting for over 90% of the total fatty acid content. Among the sixteen oat cultivars, oleic acid (C18:1) was the most abundant fatty acid, except in cultivar 22#. The oat oils were particularly rich in monounsaturated oleic acid (C18:1) and polyunsaturated linoleic acid (C18:2), which constituted approximately 40.89 ± 0.02% and 38.53 ± 0.01% of the total oil content, respectively. This composition indicated that oat oil was a valuable source of unsaturated fatty acids (UFA), particularly polyunsaturated fatty acids (PUFA). Previous studies have shown that PUFA was more effective than monounsaturated fatty acids (MUFA) in lowering low-density lipoprotein (LDL) cholesterol levels, contributing to cardiovascular health benefits [15,22]. Additionally, recent reports suggested that oats and oat oil could improve skin conditions, potentially due to the role of linoleic acid in maintaining epidermal hydration. This makes oat oil a promising candidate for the cosmetic industry [22], especially the cultivar 22#, which contains the most linoleic acid. It is important to note that the fatty acid composition of oat oil varies depending on the cultivar and growing environment. Overall, the distinct fatty acid profiles provide oat oil with significant bioactivity and functionality, allowing for the selection of specific cultivars for use in functional foods, nutritional supplements, or cosmetic applications based on targeted requirements.

#### 3.2.2. Triacylglycerol Compositions

TAGs are the primary lipid storage molecules in oat oil, accounting for approximately 50–60% of the total oil content (Table 2). As the main energy reserved in the kernel, TAGs play a crucial role in seed germination and early plant development [20]. A total of 19 TAG species were identified in oat oils, with five species present at relatively high levels (>10%). Among them, 1-palmitoyl-2-linoleoyl-3-oleyl-glycerol (PLO) and 1,3-dioleoyl-2-linoleoyl-glycerol (OLO) were the most abundant, ranging from 16.50–18.69% and 14.97–18.44%, respectively. Together, these two species accounted for approximately one-third of the total TAG content. Other major TAGs included 1-palmitoyl-2,3-dioleoyl-glycerol (POO, 11.00–13.45%), 1-palmitoyl-2,3-dilinoleoyl-glycerol (PLL, 7.87–13.43%) and 1,2-dilinoleoyl-3-oleoyl-glycerol (OLL, 10.89–12.80%). These major TAGs exhibited a common characteristic, which containing at least one palmitoyl or linoleoyl or oleoyl chain, indicating a correlation with the high content of palmitic acid, oleic acid, and linoleic acid found in oat oils. Similarly, the fatty acid type with a lower content was associated with a lower content of TAG species [23]. According to previous studies, TAG composition in oat oil is strongly influenced by its fatty acid profile [24]. For instance, similar to its unsaturated fatty acid composition, cultivar 9# exhibited significantly lower PLL and OLL contents compared to other cultivars. In contrast, cultivar 8# had the highest POO and OLO contents, consistent with its elevated C18:1 (oleic acid) levels. Furthermore, TAG composition varied significantly across different cultivars. Both fatty acid and TAG analyses confirmed that oat oil was a rich source of unsaturated fatty acids, further supporting its potential applications in the food, pharmaceutical, and cosmetic industries.

### 3.3. Lipid Concomitants Content

#### 3.3.1. Tocochromanols

Tocochromanols are essential fat-soluble antioxidants that play a key role in determining the quality and stability of oils. The tocopherol content in oat oils from different cultivars is presented in Table 3. Three tocochromanols homologs—α-tocopherol, β-tocopherol, and α-tocotrienol—were detected in some oat cultivars, with variations in both content and composition among different samples. The total tocochromanols content ranged from 4.28 ± 0.18 mg/kg (cultivar 9#) to 124.83 ± 5.02 mg/kg (cultivar 22#), with cultivar 22# exhibiting the highest α-tocopherol content. Compared to oat oil from other countries such as the U.S.A. (19–30.3 mg/kg) and Hungary (15–48 mg/kg) [7], the total tocopherol contents in Chinese oats from this study were generally slightly higher. In some oat oil samples, α-tocopherol, β-tocopherol, and α-tocotrienol were below the detection limit. While γ-tocopherol and δ-tocopherol were not detected in oat oil, the α-tocotrienol content was higher than that found in wheat germ oil [25,26]. Notably, α-tocotrienol has been reported to exhibit higher antioxidant activity than tocopherols [27], and α-tocopherol demonstrates greater antioxidant efficacy than γ-tocopherol [23]. Additionally, studies suggested that α-tocotrienol played a crucial role in preventing lipid peroxidation and may have neuroprotective effects, potentially aiding in the treatment or prevention of Alzheimer’s disease [28]. Severe tocopherol deficiency can lead to ataxia, peripheral neuropathy, muscle weakness, and retinal damage [29]. Tocochromanols could reduce the serum cholesterol concentration and also have the ability to inhibit the growth of cancer cells [7]. These findings underscore the critical role of tocochromanols in enhancing the bioactivity and nutritional quality of oat oil.

#### 3.3.2. Phytosterols

Phytosterols are bioactive compounds widely recognized for their nutritional and functional benefits in fats and oils [30]. Among them, β-sitosterol has been shown to influence cell growth and gene expression in breast cancer cells [31]. Fourteen phytosterols were identified and quantified across 16 oat cultivars, with their profiles presented in Table 4. The total phytosterol content varied significantly across different cultivars, ranging from 3380.94 ± 11.19 mg/kg to 5735.96 ± 4.80 mg/kg. Although there were differences in phytosterol content among cultivars, these variations were not statistically significant. The predominant phytosterols included campesterol, stigmasterol, β-sitosterol, ∆5-avenasterol, and ∆7-avenasterol, collectively accounting for more than 85% of the total phytosterol content. Consistent with previous reports, β-sitosterol was the most abundant phytosterol. Notably, oat oil contained significantly higher β-sitosterol levels than many other nut oils and vegetable edible oils, including olive oil (943–1732 mg/kg) [32], walnut oil (830.1–946.2 mg/kg) [20], and macadamia oil (1431.24 mg/kg) [2], and was comparable to the level found in soybean oil (1250–2360 mg/kg) [32]. Studies have demonstrated that β-sitosterol can interfere with multiple cell signaling pathways and exert anticancer effects against various cancer cells [31]. At the same time, ∆5-avenasterol levels were also extremely high, reaching up to 5 times that of macadamia oil (239.93–275.74 mg/kg) [17] and over 10 times that of walnut oil (44.9–154.4 mg/kg) [20]. A detailed analysis indicates that oat oil is a rich source of phytosterols, offering potential health benefits. Selecting specific oat cultivars could allow for targeted applications in functional foods and nutraceuticals to optimize phytosterol intake.

#### 3.3.3. Squalenes

Squalene, similar to tocotrienols, is a potent and stable antioxidant with multiple health benefits [33]. In this study, two squalene homologs were identified across different oat cultivars, with significant variations in content (Table 3). The squalene content ranged from 17.39 ± 0.35 mg/kg (cultivar 18#) to 59.33 ± 0.15 mg/kg (cultivar 8#), while squalane content ranged from 28.45 ± 0.14 mg/kg (cultivar 1#) to 34.38 ± 0.22 mg/kg (cultivar 5#). The squalene content in oat oil was lower than that found in vegetable edible oil oils such as peanut oil (98.30 mg/kg) [34], and walnut oil (11.00 mg/Kg) [20]. Squalene has been reported to reduce serum cholesterol and triglyceride levels, inhibit tumor formation, and lower the incidence of various cancers [35]. These findings suggest that oat oil, which is naturally rich in squalene, may contribute to improved cardiovascular health and cancer prevention.

#### 3.3.4. Polyphenols

Polyphenols are significant lipid-soluble bioactive compounds that contribute to the health benefits of edible oils [36]. As shown in Table 3, the total polyphenol content varied considerably among different oat cultivars, ranging from 255.47 ± 15.56 mg/kg (cultivar 1#) to 513.99 ± 10.96 mg/kg (cultivar 3#). The polyphenol content in oat oil was notably higher than that found in several other edible oils, including corn oil (18.65 mg/kg), rice bran oil (56.32 mg/kg), safflower oil (231.40 mg/kg), pumpkin seed oil (128.84 mg/kg) [37], and macadamia oil (123.40 mg/kg) [2]. Cultivar 3# exhibited nearly twice the polyphenol content of cultivar 1#, highlighting significant genetic variation in polyphenol accumulation. Polyphenols in oat oil enhance antioxidant activity and contribute to various health benefits, including anti-inflammatory and cardioprotective effects. These results suggest that oat oil is a valuable source of natural antioxidants and could be utilized in functional food formulations and nutraceutical applications.

### 3.4. Antioxidant Capacity and Correlations Between Antioxidant Capacity and Lipid Concomitants

The antioxidant capacities of oat oils, as determined by DPPH, FRAP, and ABTS assays, were presented in Table 3. Significant differences were observed among the oat oil samples. These discrepancies highlight the necessity of employing multiple radical scavenging capacity assays, as each assay is based on distinct chemical mechanisms and may capture different aspects of antioxidant properties [38]. Specifically, DPPH and ABTS assays rely on single-electron transfer mechanisms for free radical scavenging, whereas the FRAP assay does not involve free radical scavenging [38,39].

Among the three assays, the antioxidant capacity values ranged from 154.34–189.80 µmol/kg for DPPH, 834.32–2746.09 µmol/kg for FRAP, and 124.40–343.97 µmol/kg for ABTS. Notably, the antioxidant capacity of oat oil was higher than that of rice bran oil and macadamia oil [15,40], indicating its potential as a functional oil with strong antioxidative properties. Among the cultivars, sample 3# exhibited the highest antioxidant capacity, whereas cultivar 1# had the lowest. However, the trends in antioxidant capacity varied slightly among other cultivars, suggesting that different oat varieties possess distinct antioxidant profiles [35]. According to previous studies, variations in antioxidant capacity may be attributed to differences in polyphenol, squalene, and phytosterol content, among other bioactive compounds. These discrepancies further suggest that the antioxidant capacity of oat oil is not solely determined by the concentration of a single compound but also by the specific types and interactions of antioxidant substances [41,42]. Therefore, it is crucial to elucidate the relationship between antioxidant capacity and lipid concomitants in oat oil. Although this study does not focus on oats for industrial extraction, it is evident that oat oil, as a high-quality, functional oil rich in antioxidants, has potential applications as functional supplements or food additives in the food, pharmaceutical, and feed industries [5].

**Figure 3:**
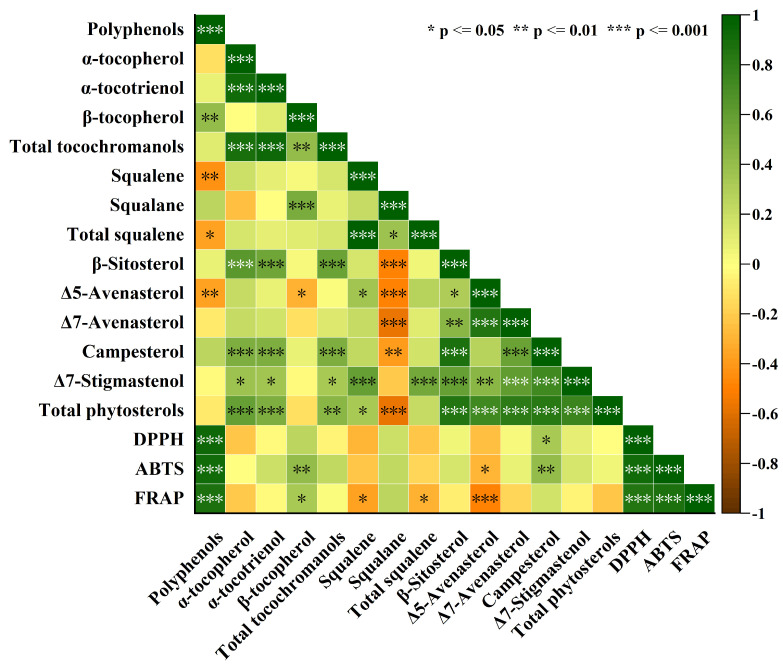
The heatmap of correlations between lipid concomitants and antioxidant capacity (* is *p* ≤ 0.05, ** is *p* ≤ 0.01 and *** is *p* ≤ 0.001).

Pearson correlation analysis (Figure 3) revealed a strong positive correlation between the bioactive components and antioxidant capacities of oat oil. Several key bioactive components—polyphenols, squalene, and ∆5-avenasterol—exhibited significant correlations with DPPH, FRAP, and ABTS assays to varying degrees. Among them, polyphenol content showed the most substantial correlation with antioxidant capacity, with correlation coefficients of *R* = 0.924 for DPPH, *R* = 0.906 for ABTS, and *R* = 0.874 for FRAP. These findings were consistent with those reported by Shuai et al. [2] (*R* = 0.939, 0.938, 0.965, respectively) and Gao et al. [20] (*R* = 0.900, 0.816, 0.902, respectively). Additionally, campesterol and *β*-tocopherol also demonstrated positive correlations with antioxidant capacity. These results indicated that polyphenols, squalene, and campesterol were the primary contributors to the antioxidant properties of oat oil. Consequently, selecting oat cultivars based on their bioactive compound profiles can help optimize their use for specific functional applications, such as in health foods and nutraceuticals.

### 3.5. Hierarchical Cluster Analysis

**Figure 4:**
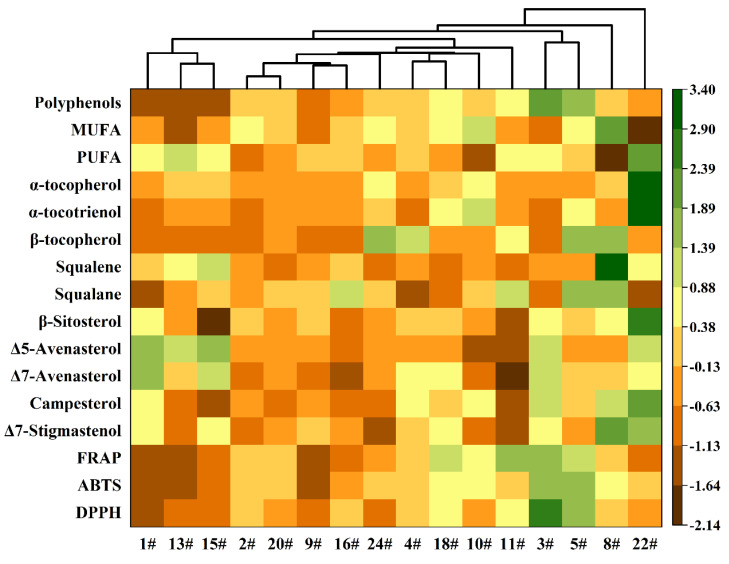
Hierarchical cluster analysis of oat oils.

In this study, twenty-one variables were used for statistical assessment, including fatty acids, triacylglycerols, lipid concomitants, and antioxidant parameters. Hierarchical Cluster Analysis (HCA) was employed to evaluate the similarities among oat oil samples based on these variables. All variables were performed using agglomerative hierarchical clustering with group average linkage, employing Euclidean distance as the distance type. The clustering heatmap shown in Figure 4 illustrates the hierarchical relationships based on the similarity of the samples. When a distance threshold of 6 was selected, the tree structure of the cluster analysis divided the samples into five main groups. These groups were clearly distinguished as follows: one group rich in phytosterols (cultivar 1#, cultivar 13#, and cultivar 15#), another with high polyphenols and strong antioxidant capacity (cultivar 3# and cultivar 5#), a group with high MUFA, triacylglycerol, and squalene content (cultivar 8#), a group with high PUFA and tocochromanol (cultivar 22#), and a routine group. Similar trends were observed among the features of 16 oat cultivars (Table S1), demonstrating a correlation between certain functional compounds and cultivar feature/phenotype characteristics. The results from this study demonstrated that oat cultivars have a significant impact on the chemical compositions, nutritional quality, and antioxidant capacity of oat oil. These findings support the potential development of oat oil for health care and cosmetic applications, and also provide valuable insights for selecting specific cultivars for breeding and processing purposes.

## 4. Conclusions

The present study provides valuable guidance for oat breeding programs in China. All analyzed oat cultivars were found to be rich in MUFA and PUFA. The primary triacylglycerols identified in oat oil were PLO, OLO, POO, PLL, and OLL. Significant variations in lipid concomitants (e.g., tocopherols and squalene) were observed among different cultivars. Notably, the detailed compositional profiles of triglycerides and phytosterols in 16 oat oils from China were reported for the first time. Moreover, a strong positive correlation was established between polyphenol content and antioxidant capacity, indicating that certain bioactive compounds play a crucial role in enhancing the oxidative stability of oat oil. Furthermore, HCA revealed that different oat cultivars can be classified into distinct groups based on their chemical composition and antioxidant properties. Given the rapid expansion of oat cultivation in China, it is essential to consider oil separation strategies based on specific oat cultivars during the fractionation process to maximize industrial utilization.

## Figures and Tables

**Table 1:** Fatty acid compositions (%) of oils extracted from sixteen oat cultivars ^1^.

Name	C14:0 *	C16:0	C16:1	C17:0	C18:0	C18:1	C18:2	C18:3	C20:0	C20:1	C22:0	C23:0	C24:0
1#	0.16 ± 0.00 ^i^	13.38 ± 0.06 ^j^	0.17 ± 0.00 ^cd^	0.05 ± 0.00 ^cd^	2.51 ± 0.01 ^e^	40.47 ± 0.05 ^j^	39.87 ± 0.02 ^c^	1.00 ± 0.00 ^h^	0.35 ± 0.00 ^d^	1.24 ± 0.00 ^cde^	0.20 ± 0.00 ^b^	0.18 ± 0.01 ^b^	0.42 ± 0.01 ^h^
2#	0.20 ± 0.00 ^f^	14.98 ± 0.14 ^cd^	0.14 ± 0.00 ^ef^	0.05 ± 0.00 ^cd^	2.13 ± 0.01 ^h^	41.63 ± 0.07 ^f^	37.49 ± 0.05 ^l^	0.99 ± 0.00 ^h^	0.33 ± 0.00 ^e^	1.22 ± 0.02 ^def^	0.18 ± 0.01 ^c^	0.29 ± 0.01 ^ab^	0.45 ± 0.01 ^gh^
3#	0.22 ± 0.00 ^b^	14.18 ± 0.04 ^h^	0.17 ± 0.00 ^c^	0.05 ± 0.00 ^fg^	2.59 ± 0.00 ^d^	39.48 ± 0.04 ^m^	39.48 ± 0.05 ^e^	1.19 ± 0.02 ^d^	0.39 ± 0.00 ^b^	1.29 ± 0.00 ^b^	0.22 ± 0.00 ^a^	0.20 ± 0.02 ^a^	0.55 ± 0.04 ^cd^
4#	0.21 ± 0.00 ^d^	13.99 ± 0.02 ^i^	0.14 ± 0.00 ^ef^	0.05 ± 0.00 ^ef^	2.68 ± 0.00 ^c^	40.65 ± 0.04 ^i^	38.62 ± 0.02 ^h^	1.10 ± 0.01 ^ef^	0.40 ± 0.00 ^ab^	1.20 ± 0.00 ^fgh^	0.22 ± 0.00 ^a^	0.15 ± 0.00 ^cd^	0.60 ± 0.02 ^ab^
5#	0.15 ± 0.00 ^j^	13.16 ± 0.01 ^k^	0.15 ± 0.00 ^de^	0.05 ± 0.00 ^de^	2.48 ± 0.00 ^f^	42.00 ± 0.03 ^e^	38.91 ± 0.01 ^f^	1.05 ± 0.00 ^fg^	0.32 ± 0.00 ^ef^	1.19 ± 0.00 ^gh^	0.18 ± 0.01 ^cd^	——	0.38 ± 0.00 ^i^
8#	0.14 ± 0.00 ^jk^	13.24 ± 0.00 ^k^	0.09 ± 0.00 ^k^	0.06 ± 0.00 ^ab^	3.41 ± 0.00 ^b^	45.22 ± 0.04 ^a^	34.13 ± 0.02 ^n^	1.40 ± 0.01 ^b^	0.40 ± 0.00 ^ab^	1.19 ± 0.00 ^h^	0.16 ± 0.00 ^e^	0.08 ± 0.01 ^g^	0.47 ± 0.04 ^fg^
9#	0.25 ± 0.00 ^a^	15.05 ± 0.08 ^bc^	0.16 ± 0.00 ^d^	0.06 ± 0.00 ^a^	2.50 ± 0.00 ^e^	39.53 ± 0.03 ^m^	38.70 ± 0.02 ^g^	1.04 ± 0.05 ^g^	0.41 ± 0.00 ^a^	1.25 ± 0.00 ^c^	0.22 ± 0.00 ^a^	0.16 ± 0.01 ^c^	0.64 ± 0.00 ^a^
10#	0.14 ± 0.00 ^k^	13.97 ± 0.04 ^i^	0.13 ± 0.00 ^f^	0.04 ± 0.00 ^g^	3.57 ± 0.00 ^a^	43.44 ± 0.02 ^b^	35.34 ± 0.02 ^m^	1.17 ± 0.01 ^d^	0.38 ± 0.00 ^c^	1.08 ± 0.00 ^j^	0.14 ± 0.00 ^gh^	0.06 ± 0.00 ^hi^	0.58 ± 0.03 ^bc^
11#	0.18 ± 0.00 ^g^	14.44 ± 0.03 ^g^	0.14 ± 0.00 ^e^	0.05 ± 0.00 ^fg^	1.82 ± 0.00 ^m^	40.30 ± 0.04 ^k^	39.71 ± 0.05 ^d^	1.16 ± 0.00 ^d^	0.26 ± 0.00 ^i^	1.16 ± 0.01 ^i^	0.14 ± 0.01 ^fg^	0.11 ± 0.00 ^f^	0.58 ± 0.02 ^bc^
13#	0.22 ± 0.00 ^b^	14.91 ± 0.00 ^de^	0.20 ± 0.00 ^b^	0.05 ± 0.00 ^cd^	2.15 ± 0.00 ^g^	38.34 ± 0.03 ^n^	40.68 ± 0.05 ^b^	1.06 ± 0.01 ^fg^	0.31 ± 0.00 ^fg^	1.21 ± 0.00 ^fgh^	0.18 ± 0.01 ^c^	0.13 ± 0.00 ^e^	0.61 ± 0.01 ^ab^
15#	0.18 ± 0.00 ^g^	14.14 ± 0.06 ^h^	0.15 ± 0.02 ^e^	0.06 ± 0.00 ^ab^	2.48 ± 0.04 ^f^	40.02 ± 0.01 ^l^	39.91 ± 0.13 ^c^	1.18 ± 0.02 ^d^	0.28 ± 0.02 ^h^	1.00 ± 0.03 ^k^	0.13 ± 0.00 ^h^	0.09 ± 0.00 ^fg^	0.47 ± 0.01 ^fg^
16#	0.20 ± 0.00 ^e^	14.82 ± 0.01 ^e^	0.14 ± 0.00 ^e^	0.05 ± 0.00 ^ef^	1.99 ± 0.00 ^k^	40.83 ± 0.05 ^h^	38.48 ± 0.01 ^i^	1.11 ± 0.01 ^e^	0.31 ± 0.00 ^g^	1.24 ± 0.01 ^cde^	0.17 ± 0.01 ^d^	0.14 ± 0.03 ^de^	0.45 ± 0.02 ^gh^
18#	0.16 ± 0.00 ^i^	14.37 ± 0.04 ^g^	0.15 ± 0.00 ^e^	0.04 ± 0.00 ^h^	1.76 ± 0.00 ^n^	42.11 ± 0.00 ^d^	37.95 ± 0.00 ^j^	1.07 ± 0.01 ^fg^	0.29 ± 0.00 ^h^	1.37 ± 0.01 ^a^	0.18 ± 0.00 ^cd^	0.13 ± 0.01 ^e^	0.42 ± 0.00 ^h^
20#	0.20 ± 0.00 ^de^	15.13 ± 0.00 ^b^	0.13 ± 0.00 ^f^	0.06 ± 0.00 ^a^	2.04 ± 0.00 ^j^	40.96 ± 0.02 ^g^	37.95 ± 0.03 ^j^	1.24 ± 0.01 ^cd^	0.32 ± 0.00 ^ef^	1.21 ± 0.01 ^efg^	0.17 ± 0.01 ^cd^	0.09 ± 0.00 ^g^	0.49 ± 0.04 ^ef^
22#	0.21 ± 0.00 ^c^	15.55 ± 0.00 ^a^	0.37 ± 0.00 ^a^	0.04 ± 0.00 ^h^	1.46 ± 0.00 ^o^	37.03 ± 0.02 ^o^	41.60 ± 0.01 ^a^	1.69 ± 0.07 ^a^	0.69 ± 0.07 ^j^	1.23 ± 0.01 ^cde^	0.14 ± 0.00 ^fg^	0.06 ± 0.00 ^i^	0.53 ± 0.04 ^de^
24#	0.17 ± 0.00 ^h^	14.55 ± 0.15 ^f^	0.11 ± 0.00 ^g^	0.05 ± 0.00 ^bc^	1.97 ± 0.00 ^l^	42.32 ± 0.00 ^c^	37.68 ± 0.01 ^k^	0.93 ± 0.00 ^i^	0.29 ± 0.01 ^h^	1.24 ± 0.03 ^cd^	0.15 ± 0.00 ^ef^	0.08 ± 0.00 ^gh^	0.55 ± 0.00 ^cd^

^1^ Values were means ± SD. Different letters in a column indicate significant differences (*p* < 0.05). * C14:0, myristic acid; C16:0, palmitic acid; C16:1, palmitoleic acid; C18:0, stearic acid; C18:1, oleic acid; C18:2, linoleic acid; C18:3, linolenic acid; C20:0, eicosanoic acid; C20:1, eicosenoic acid; C22:0, docosanoic acid; C23:0, tricosanoic acid; C24:0, lignoceric acid.

**Table 2:** Triacylglycerols (% (*w*/*w*)) of oils extracted from sixteen oat cultivars ^1^.

Name	1#	2#	3#	4#	5#	8#	9#	10#	11#	13#	15#	16#	18#	20#	22#	24#
PPP *	0.44 ± 0.01 ^f^	0.50 ± 0.01 ^e^	0.75 ± 0.00 ^a^	0.51 ± 0.00 ^d^	0.22 ± 0.00 ^l^	0.33 ± 0.00 ^i^	0.58 ± 0.01 ^c^	0.31 ± 0.00 ^j^	0.38 ± 0.00 ^g^	0.60 ± 0.01 ^b^	0.31 ± 0.00 ^j^	0.36 ± 0.00 ^h^	0.36 ± 0.00 ^h^	0.34 ± 0.01 ^i^	0.27 ± 0.00 ^k^	0.38 ± 0.00 ^g^
MOP	0.18 ± 0.00 ^gh^	0.21 ± 0.01 ^e^	0.24 ± 0.00 ^b^	0.23 ± 0.00 ^c^	0.16 ± 0.00 ^k^	0.17 ± 0.00 ^i^	0.27 ± 0.00 ^a^	0.16 ± 0.00 ^j^	0.20 ± 0.00 ^f^	0.23 ± 0.00 ^c^	0.18 ± 0.01 ^hi^	0.21 ± 0.00 ^e^	0.17 ± 0.00 ^j^	0.21 ± 0.00 ^e^	0.17 ± 0.05 ^d^	0.18 ± 0.00 ^g^
MLP	0.19 ± 0.02 ^c^	0.22 ± 0.01 ^b^	0.23 ± 0.00 ^b^	0.22 ± 0.00 ^b^	0.14 ± 0.00 ^e^	0.13 ± 0.00 ^f^	0.26 ± 0.00 ^a^	0.14 ± 0.00 ^ef^	0.19 ± 0.02 ^c^	0.23 ± 0.00 ^b^	0.17 ± 0.00 ^d^	0.19 ± 0.00 ^c^	0.14 ± 0.00 ^e^	0.19 ± 0.00 ^c^	0.22 ± 0.00 ^b^	0.15 ± 0.00 ^e^
PPS	0.22 ± 0.00 ^e^	0.18 ± 0.00 ^f^	0.34 ± 0.00 ^a^	0.24 ± 0.00 ^d^	0.13 ± 0.00 ^jk^	0.29 ± 0.00 ^b^	0.24 ± 0.00 ^d^	0.27 ± 0.01 ^c^	0.12 ± 0.01 ^k^	0.24 ± 0.00 ^d^	0.16 ± 0.00 ^g^	0.14 ± 0.00 ^hi^	0.13 ± 0.01 ^ij^	0.14 ± 0.01 ^hi^	0.08 ± 0.00 ^l^	0.14 ± 0.00 ^h^
POP	4.60 ± 0.03 ^g^	5.49 ± 0.05 ^a^	5.10 ± 0.00 ^d^	5.04 ± 0.11 ^de^	4.28 ± 0.04 ^j^	5.01 ± 0.00 ^e^	5.40 ± 0.01 ^b^	5.20 ± 0.03 ^c^	4.98 ± 0.00 ^e^	4.79 ± 0.02 ^f^	4.52 ± 0.00 ^h^	5.03 ± 0.02 ^de^	4.77 ± 0.05 ^f^	5.21 ± 0.02 ^c^	4.45 ± 0.07 ^i^	5.02 ± 0.00 ^e^
MOO	0.18 ± 0.00 ^de^	0.22 ± 0.00 ^b^	0.23 ± 0.00 ^ab^	0.23 ± 0.01 ^a^	0.17 ± 0.01 ^e^	0.19 ± 0.00 ^c^	0.22 ± 0.00 ^ab^	0.12 ± 0.05 ^e^	0.20 ± 0.01 ^c^	0.14 ± 0.06 ^c^	0.17 ± 0.01 ^e^	0.14 ± 0.06 ^cd^	0.17 ± 0.01 ^e^	0.21 ± 0.00 ^b^	0.17 ± 0.00 ^e^	0.19 ± 0.00 ^cd^
PLP	4.97 ± 0.00 ^f^	5.53 ± 0.05 ^c^	5.60 ± 0.01 ^c^	5.20 ± 0.04 ^e^	4.34 ± 0.02 ^i^	3.75 ± 0.02 ^j^	5.95 ± 0.00 ^b^	4.42 ± 0.07 ^i^	5.30 ± 0.00 ^d^	5.91 ± 0.12 ^b^	5.29 ± 0.01 ^d^	5.21 ± 0.11 ^e^	4.78 ± 0.02 ^g^	5.24 ± 0.01 ^de^	6.47 ± 0.02 ^a^	4.62 ± 0.00 ^h^
MLO	0.39 ± 0.03 ^f^	0.39 ± 0.01 ^f^	0.50 ± 0.00 ^c^	0.45 ± 0.00 ^d^	0.37 ± 0.01 ^g^	0.30 ± 0.00 ^j^	0.52 ± 0.00 ^b^	0.32 ± 0.01 ^i^	0.40 ± 0.01 ^f^	0.52 ± 0.01 ^b^	0.43 ± 0.01 ^e^	0.42 ± 0.01 ^e^	0.35 ± 0.01 ^h^	0.42 ± 0.00 ^e^	0.69 ± 0.05 ^a^	0.36 ± 0.00 ^h^
POS	1.24 ± 0.00 ^g^	1.18 ± 0.01 ^h^	1.32 ± 0.01 ^e^	1.47 ± 0.01 ^c^	1.28 ± 0.01 ^f^	2.08 ± 0.01 ^b^	1.35 ± 0.01 ^d^	2.18 ± 0.01 ^a^	0.97 ± 0.00 ^l^	1.08 ± 0.01 ^k^	1.10 ± 0.00 ^j^	1.08 ± 0.00 ^k^	0.98 ± 0.00 ^l^	1.16 ± 0.01 ^i^	0.68 ± 0.01 ^m^	1.09 ± 0.01 ^k^
POO	11.55 ± 0.01 ^k^	13.07 ± 0.09 ^b^	11.53 ± 0.02 ^k^	11.91 ± 0.10 ^i^	11.99 ± 0.02 ^h^	13.45 ± 0.01 ^a^	12.05 ± 0.01 ^h^	12.95 ± 0.01 ^c^	12.25 ± 0.01 ^g^	11.21 ± 0.04 ^l^	11.67 ± 0.01 ^j^	12.48 ± 0.03 ^f^	12.71 ± 0.05 ^de^	12.74 ± 0.02 ^d^	11.00 ± 0.07 ^m^	12.66 ± 0.00 ^e^
PLS	1.30 ± 0.01 ^e^	1.13 ± 0.01 ^gh^	1.36 ± 0.01 ^c^	1.34 ± 0.01 ^d^	1.16 ± 0.00 ^g^	1.44 ± 0.01 ^b^	1.29 ± 0.01 ^e^	1.57 ± 0.00 ^a^	0.99 ± 0.01 ^kl^	1.26 ± 0.00 ^f^	1.12 ± 0.00 ^h^	1.07 ± 0.01 ^i^	0.98 ± 0.00 ^l^	1.02 ± 0.01 ^j^	1.01 ± 0.05 ^jk^	0.92 ± 0.01 ^m^
PLO	18.17 ± 0.14 ^de^	18.31 ± 0.12 ^c^	18.09 ± 0.03 ^e^	17.95 ± 0.00 ^f^	17.51 ± 0.08 ^g^	16.50 ± 0.01 ^i^	18.31 ± 0.03 ^c^	17.30 ± 0.09 ^h^	18.43 ± 0.01 ^b^	18.69 ± 0.07 ^a^	18.14 ± 0.02 ^de^	18.06 ± 0.01 ^e^	18.16 ± 0.08 ^de^	18.23 ± 0.03 ^cd^	18.59 ± 0.00 ^a^	18.44 ± 0.01 ^b^
PLL	10.36 ± 0.01 ^j^	10.53 ± 0.06 ^i^	10.62 ± 0.03 ^h^	10.58 ± 0.09 ^hi^	10.72 ± 0.02 ^g^	7.87 ± 0.08 ^m^	11.21 ± 0.01 ^d^	9.17 ± 0.02 ^l^	11.20 ± 0.01 ^d^	11.65 ± 0.03 ^b^	11.46 ± 0.03 ^c^	11.01 ± 0.02 ^e^	10.30 ± 0.06 ^j^	10.86 ± 0.03 ^f^	13.73 ± 0.03 ^a^	9.73 ± 0.03 ^k^
SOO	1.46 ± 0.00 ^g^	1.49 ± 0.01 ^f^	1.50 ± 0.00 ^ef^	1.69 ± 0.00 ^c^	1.56 ± 0.01 ^d^	2.25 ± 0.00 ^a^	1.51 ± 0.01 ^e^	2.14 ± 0.01 ^b^	1.24 ± 0.00 ^l^	1.25 ± 0.00 ^l^	1.29 ± 0.02 ^k^	1.37 ± 0.01 ^j^	1.27 ± 0.01 ^k^	1.44 ± 0.00 ^h^	0.93 ± 0.00 ^m^	1.41 ± 0.01 ^i^
OOO	8.17 ± 0.02 ^h^	8.49 ± 0.34 ^fg^	7.70 ± 0.02 ^i^	7.57 ± 0.36 ^i^	9.17 ± 0.00 ^bc^	10.82 ± 0.06 ^a^	7.71 ± 0.01 ^i^	9.38 ± 0.06 ^b^	8.28 ± 0.02 ^gh^	7.14 ± 0.03 ^j^	8.20 ± 0.01 ^h^	8.68 ± 0.01 ^ef^	9.12 ± 0.03 ^c^	8.87 ± 0.03 ^de^	7.20 ± 0.01 ^j^	8.98 ± 0.06 ^cd^
SOL	2.16 ± 0.01 ^c^	1.46 ± 0.35 ^f^	2.18 ± 0.00 ^c^	1.83 ± 0.37 ^c^	1.93 ± 0.01 ^e^	2.53 ± 0.02 ^a^	2.05 ± 0.10 ^d^	2.49 ± 0.01 ^b^	1.64 ± 0.02 ^j^	2.01 ± 0.02 ^d^	1.74 ± 0.00 ^g^	1.76 ± 0.02 ^g^	1.66 ± 0.02 ^ij^	1.70 ± 0.02 ^h^	1.50 ± 0.01 ^k^	1.68 ± 0.02 ^hi^
OLO	16.87 ± 0.01 ^e^	15.76 ± 0.18 ^h^	15.90 ± 0.04 ^h^	16.94 ± 0.28 ^e^	17.97 ± 0.09 ^b^	18.44 ± 0.20 ^a^	15.14 ± 0.04 ^j^	17.27 ± 0.08 ^d^	16.25 ± 0.07 ^g^	15.50 ± 0.03 ^i^	16.67 ± 0.04 ^f^	16.22 ± 0.03 ^g^	17.16 ± 0.07 ^d^	16.13 ± 0.01 ^g^	14.97 ± 0.03 ^j^	17.49 ± 0.01 ^c^
OLL	12.80 ± 0.06 ^a^	11.57 ± 0.07 ^f^	12.32 ± 0.03 ^b^	12.03 ± 0.06 ^d^	11.95 ± 0.10 ^de^	11.00 ± 0.14 ^i^	11.42 ± 0.02 ^g^	10.89 ± 0.05 ^j^	12.15 ± 0.05 ^c^	12.72 ± 0.08 ^a^	12.31 ± 0.07 ^b^	11.85 ± 0.03 ^e^	12.26 ± 0.04 ^b^	11.29 ± 0.03 ^h^	11.95 ± 0.03 ^de^	12.32 ± 0.03 ^b^
LLL	4.75 ± 0.15 ^e^	4.28 ± 0.03 ^g^	4.50 ± 0.02 ^f^	4.58 ± 0.01 ^f^	4.95 ± 0.00 ^c^	3.46 ± 0.00 ^i^	4.51 ± 0.01 ^f^	3.73 ± 0.04 ^h^	4.84 ± 0.01 ^d^	4.85 ± 0.03 ^d^	5.08 ± 0.02 ^b^	4.73 ± 0.02 ^e^	4.52 ± 0.01 ^f^	4.57 ± 0.01 ^f^	5.92 ± 0.13 ^a^	4.25 ± 0.05 ^g^

^1^ Values were means ± SD. Different letters in a line indicate significant differences (*p* < 0.05). * PPP: 1,2-dipalmitoyl-3-palmitoleyl-glycerol, MOP: 1-myristoyl-2-oleoyl-3-palmitoyl-glycerol, MLP: 1-myristoyl-2-linoleoyl-3-palmitoyl-glycerol, PPS: 1,2-dipalmitoyl-3-stearoyl-glycerol, POP: 1,3-dipalmitoyl-2-oleyl-glycerol, MOO: 1-myristoyl-2,3-dioleoyl-glycerol, PLP: 1-palmitoyl-2-linoleoyl-3-palmitoleyl-glycerol, MLO: 1-myristoyl-2-linoleoyl-3-oleyl-glycerol, POS: 1-palmitoleyl-2-oleyl-3-stearoyl-glycerol, POO: 1-palmitoyl-2,3-dioleoyl-glycerol, PLS: 1-palmitoleyl-2-linoleoyl-3-stearoyl-glycerol, PLO: 1-palmitoyl-2-linoleoyl-3-oleyl-glycerol, PLL: 1-palmitoyl-2,3-dilinoleoyl-glycerol, SOO: 1-stearoyl-2,3-dioleyl-glycerol, OOO: triolein, SOL: 1-stearoyl-2-oleoyl-3-linoleoyl-glycerol, OLO: 1,3-dioleoyl-2-linoleoyl-glycerol, OLL: 1,2-dilinoleoyl-3-oleoyl-glycerol, and LLL: trilinolein.

**Table 3:** The antioxidant capacity (µmol Vitamin E/kg) and lipid companion content (mg/kg of oil) of oils extracted from sixteen oat cultivars ^1^.

Name	Antioxidant Capacity (μmol/kg)			Polyphenols (mg/kg)	Total Squalanes (mg/kg)		Total Tocochromanols (mg/kg)		
	DPPH *	ABTS	FRAP		Squalane	Squalene	α-Tocopherol	α-Tocotrienol	β-Tocopherol
1#	154.34 ± 0.90 ^m^	124.40 ± 0.95 ^n^	834.32 ± 3.12 ^n^	255.47 ± 15.56 ^k^	28.45 ± 0.14 ^m^	29.33 ± 0.24 ^f^	——	——	——
2#	165.22 ± 0.00 ^g^	232.06 ± 1.25 ^f^	1908.89 ± 1.18 ^f^	351.84 ± 4.14 ^fg^	30.33 ± 0.27 ^h^	26.60 ± 0.26 ^g^	——	——	——
3#	189.80 ± 0.20 ^a^	343.97 ± 0.95 ^a^	2746.09 ± 3.11 ^a^	513.99 ± 10.96 ^a^	29.57 ± 0.04 ^j^	21.55 ± 0.01 ^k^	——	——	——
4#	168.90 ± 0.00 ^d^	236.94 ± 0.62 ^e^	1852.99 ± 11.23 ^g^	363.35 ± 5.78 ^ef^	28.89 ± 0.04 ^l^	23.45 ± 0.27 ^j^	——	——	20.73 ± 0.18 ^d^
5#	181.06 ± 0.00 ^b^	336.38 ± 0.36 ^b^	2538.68 ± 7.34 ^c^	492.44 ± 0.73 ^b^	34.38 ± 0.22 ^a^	21.83 ± 0.45 ^k^	2.52 ± 0.01 ^e^	22.31 ± 1.13 ^c^	30.50 ± 0.30 ^b^
8#	168.15 ± 0.20 ^e^	259.90 ± 1.57 ^d^	1936.68 ± 13.37 ^e^	358.06 ± 7.95 ^ef^	33.95 ± 0.25 ^b^	59.33 ± 0.15 ^a^	7.85 ± 0.23 ^d^	8.92 ± 1.07 ^f^	28.92 ± 0.82 ^c^
9#	158.87 ± 0.20 ^k^	134.07 ± 0.95 ^l^	914.55 ± 9.34 ^m^	291.34 ± 12.68 ^i^	31.45 ± 0.25 ^f^	25.43 ± 0.17 ^h^	——	4.28 ± 0.18 ^h^	——
10#	164.38 ± 0.20 ^de^	269.24 ± 0.63 ^c^	2202.62 ± 2.36 ^d^	364.63 ± 6.60 ^ef^	31.74 ± 0.13 ^e^	24.51 ± 0.12 ^i^	20.82 ± 0.68 ^b^	24.09 ± 0.02 ^b^	5.10 ± 0.03 ^g^
11#	170.29 ± 0.00 ^c^	218.20 ± 1.29 ^h^	2745.37 ± 2.03 ^a^	418.39 ± 6.42 ^c^	33.49 ± 0.08 ^c^	20.87 ± 0.12 ^l^	——	2.52 ± 0.36 ^i^	17.57 ± 0.03 ^e^
13#	157.96 ± 0.20 ^l^	131.03 ± 0.95 ^m^	816.14 ± 12.46 ^o^	265.69 ± 14.07 ^jk^	30.63 ± 0.11 ^g^	36.25 ± 0.46 ^c^	15.34 ± 0.62 ^c^	8.98 ± 0.45 ^f^	——
15#	158.42 ± 0.34 ^kl^	172.16 ± 0.72 ^k^	994.98 ± 14.34 ^l^	271.62 ± 12.07 ^j^	31.77 ± 0.09 ^e^	39.58 ± 0.08 ^b^	7.79 ± 0.09 ^d^	7.82 ± 0.18 ^f^	——
16#	167.72 ± 0.00 ^h^	183.91 ± 0.95 ^j^	1094.51 ± 7.15 ^k^	327.30 ± 8.76 ^h^	32.91 ± 0.07 ^d^	31.07 ± 0.46 ^e^	2.30 ± 0.01 ^e^	4.97 ± 0.50 ^gh^	——
18#	170.25 ± 0.34 ^c^	268.61 ± 0.36 ^c^	2570.06 ± 13.25 ^b^	407.32 ± 2.55 ^c^	29.93 ± 0.10 ^i^	17.39 ± 0.35 ^n^	15.53 ± 0.84 ^c^	18.08 ± 0.96 ^d^	4.28 ± 0.83 ^h^
20#	163.60 ± 0.52 ^f^	220.75 ± 0.95 ^g^	1820.92 ± 5.12 ^h^	386.96 ± 3.83 ^d^	31.25 ± 0.06 ^f^	20.05 ± 0.63 ^m^	——	6.12 ± 0.34 ^g^	4.43 ± 0.17 ^h^
22#	161.23 ± 0.00 ^i^	215.98 ± 0.36 ^i^	1137.01 ± 8.23 ^j^	337.44 ± 0.36 ^gh^	29.14 ± 0.17 ^k^	35.49 ± 0.05 ^d^	66.43 ± 2.43 ^a^	52.17 ± 2.97 ^a^	6.23 ± 0.38 ^f^
24#	159.97 ± 0.39 ^j^	215.06 ± 0.62 ^j^	1337.91 ± 12.48 ^i^	371.77 ± 11.04 ^e^	31.86 ± 0.09 ^e^	16.38 ± 0.11 ^o^	16.12 ± 0.28 ^c^	11.21 ± 0.07 ^e^	31.71 ± 0.11 ^a^

^1^ Values were means ± SD. Different letters in a column indicate significant differences (*p* < 0.05). * DPPH: 2,2-Diphenyl-1-picrylhydrazyl radical scavenging ability. ABTS: 2,2′-Azino-bis (3-ethylbenzothiazoline-6-sulfonic acid) diammonium salt radical scavenging ability. FRAP: Ferric reducing ability of plasma.

**Table 4:** Phytosterols (mg/kg) of oils extracted from sixteen oat cultivars ^1^.

Name	1#	2#	3#	4#	5#	8#	9#	10#	11#	13#	15#	16#	18#	20#	22#	24#
brassicasterol	50.06 ± 0.41 ^gh^	53.18 ± 2.73 ^g^	56.51 ± 1.89 ^f^	51.01 ± 0.13 ^gh^	63.93 ± 3.10 ^e^	73.94 ± 0.55 ^d^	45.72 ± 0.99 ^i^	60.88 ± 0.74 ^e^	46.62 ± 0.96 ^i^	48.66 ± 0.90 ^hi^	84.76 ± 0.10 ^b^	51.92 ± 1.74 ^gh^	79.73 ± 4.38 ^c^	36.44 ± 1.15 ^k^	97.27 ± 2.58 ^a^	40.95 ± 1.34 ^j^
2,4-methylene cholestadienol	67.57 ± 2.41 ^e^	50.41 ± 0.36 ^h^	90.80 ± 2.99 ^c^	61.89 ± 0.15 ^f^	61.45 ± 1.91 ^f^	103.15 ± 1.63 ^a^	55.67 ± 1.23 ^g^	85.10 ± 0.19 ^d^	50.08 ± 0.18 ^h^	68.51 ± 1.17 ^e^	96.18 ± 0.63 ^b^	46.72 ± 0.01 ^i^	69.69 ± 4.22 ^e^	43.62 ± 0.62 ^j^	104.01 ± 1.95 ^a^	42.86 ± 2.36 ^j^
Campesterol	310.32 ± 0.11 ^d^	249.17 ± 0.27 ^j^	355.27 ± 2.20 ^b^	304.02 ± 0.75 ^e^	289.67 ± 0.36 ^g^	339.91 ± 0.53 ^c^	262.22 ± 0.28 ^i^	300.40 ± 0.65 ^f^	201.54 ± 0.41 ^n^	231.81 ± 1.15 ^k^	202.035 ± 1.76 ^n^	215.07 ± 1.10 ^m^	283.99 ± 3.02 ^h^	233.42 ± 0.09 ^k^	399.11 ± 1.31 ^a^	221.22 ± 2.86 ^l^
Campesteranol	24.77 ± 0.25 ^b^	14.93 ± 0.08 ^j^	23.33 ± 0.47 ^c^	18.78 ± 0.05 ^gh^	21.52 ± 0.00 ^e^	19.03 ± 0.55 ^g^	18.37 ± 0.01 ^h^	18.71 ± 0.12 ^gh^	18.34 ± 0.41 ^h^	20.30 ± 0.09 ^f^	22.78 ± 0.16 ^d^	16.64 ± 0.33 ^i^	20.18 ± 0.13 ^f^	18.75 ± 0.24 ^gh^	32.59 ± 0.51 ^a^	18.51 ± 0.14 ^gh^
Stigmasterol	211.07 ± 9.49 ^b^	130.32 ± 0.22 ^h^	174.86 ± 3.88 ^cd^	137.05 ± 0.34 ^gh^	167.38 ± 3.91 ^d^	176.58 ± 1.73 ^c^	137.91 ± 5.82 ^g^	156.11 ± 0.64 ^e^	137.84 ± 1.71 ^g^	174.44 ± 10.10 ^cd^	181.90 ± 0.13 ^c^	140.99 ± 1.76 ^g^	174.79 ± 0.35 ^cd^	148.26 ± 2.99 ^f^	281.66 ± 0.16 ^a^	117.80 ± 1.66 ^i^
*Δ^7^*-campesterol	17.31 ± 0.00 ^d^	9.33 ± 0.43 ^g^	15.30 ± 0.49 ^e^	6.86 ± 0.02 ^h^	16.18 ± 2.17 ^de^	26.42 ± 1.89 ^a^	16.12 ± 1.18 ^de^	16.15 ± 0.03 ^de^	10.30 ± 0.70 ^fg^	8.95 ± 0.73 ^g^	18.98 ± 0.46 ^c^	9.68 ± 1.12 ^fg^	16.23 ± 0.17 ^de^	11.20 ± 0.00 ^f^	21.33 ± 1.01 ^b^	14.68 ± 1.30 ^e^
*Δ^5,23^*-Stigmastadienol	87.38 ± 1.44 ^bc^	78.16 ± 1.05 ^e^	66.35 ± 0.44 ^ij^	71.35 ± 0.17 ^g^	86.08 ± 3.12 ^c^	69.67 ± 0.46 ^gh^	66.17 ± 0.53 ^ij^	69.82 ± 1.17 ^gh^	83.67 ± 1.23 ^d^	65.29 ± 1.02 ^j^	68.91 ± 0.95 ^h^	74.03 ± 1.36 ^f^	76.98 ± 1.42 ^e^	106.53 ± 1.10 ^a^	89.31 ± 2.04 ^b^	68.12 ± 1.62 ^hi^
Clerosterol	105.83 ± 0.39 ^b^	67.70 ± 1.51 ^jk^	88.67 ± 2.33 ^d^	75.14 ± 0.18 ^g^	79.98 ± 1.06 ^f^	69.53 ± 0.41 ^ij^	91.55 ± 2.19 ^c^	66.76 ± 3.25 ^k^	61.87 ± 0.57 ^l^	82.78 ± 0.15 ^e^	104.72 ± 0.94 ^b^	72.24 ± 0.08 ^h^	82.99 ± 2.63 ^e^	67.43 ± 1.00 ^jk^	117.44 ± 1.13 ^a^	70.37 ± 1.24 ^hi^
*β*-Sitosterol	1868.90 ± 0.65 ^b^	1670.81 ± 6.38 ^f^	1846.43 ± 17.29 ^c^	1697.75 ± 4.22 ^e^	1649.92 ± 2.52 ^g^	1812.32 ± 12.66 ^d^	1668.64 ± 0.23 ^f^	1566.64 ± 5.29 ^i^	1359.11 ± 3.66 ^k^	1561.59 ± 3.22 ^i^	1253.79 ± 0.98 ^l^	1426.99 ± 4.65 ^j^	1666.63 ± 1.49 ^f^	1624.31 ± 6.40 ^h^	2368.93 ± 1.06 ^a^	1632.73 ± 6.50 ^h^
*β*-Stiostanol	71.59 ± 1.28 ^a^	34.82 ± 0.93 ^g^	43.23 ± 0.98 ^e^	42.67 ± 0.11 ^e^	45.88 ± 0.53 ^d^	61.34 ± 1.95 ^b^	38.26 ± 1.15 ^f^	49.44 ± 0.63 ^c^	28.31 ± 0.99 ^h^	46.32 ± 2.18 ^d^	42.66 ± 1.11 ^e^	30.40 ± 1.14 ^h^	40.46 ± 0.00 ^f^	37.82 ± 0.94 ^f^	72.64 ± 0.40 ^a^	49.45 ± 0.92 ^c^
*Δ^5^*-Avenasterol	1379.29 ± 5.12 ^a^	1019.58 ± 10.41 ^g^	1233.18 ± 15.60 ^c^	1058.86 ± 2.63 ^de^	1050.45 ± 4.78 ^ef^	1063.54 ± 2.57 ^d^	998.10 ± 8.96 ^h^	891.35 ± 4.30 ^j^	852.39 ± 1.38 ^k^	1256.77 ± 3.12 ^b^	1382.55 ± 0.25 ^a^	953.95 ± 1.10 ^i^	1017.18 ± 2.63 ^g^	995.43 ± 6.13 ^h^	1247.56 ± 11.06 ^b^	1040.89 ± 9.31 ^f^
*Δ^5,24^*-Stigmastadienol	150.56 ± 3.20 ^c^	118.11 ± 0.09 ^f^	171.35 ± 1.62 ^b^	126.21 ± 0.31 ^e^	154.28 ± 4.30 ^c^	88.09 ± 3.17 ^h^	126.41 ± 1.33 ^e^	125.60 ± 2.22 ^e^	90.12 ± 2.20 ^h^	140.74 ± 2.45 ^d^	140.51 ± 3.38 ^d^	107.90 ± 1.84 ^g^	139.96 ± 9.39 ^d^	101.16 ± 9.15 ^g^	180.09 ± 1.01 ^a^	102.72 ± 0.14 ^g^
*Δ^7^*-Stigmastenol	190.86 ± 1.18 ^de^	155.95 ± 1.48 ^h^	198.83 ± 0.17 ^c^	184.90 ± 0.46 ^e^	176.26 ± 4.83 ^g^	234.41 ± 1.60 ^a^	186.65 ± 3.22 ^e^	157.69 ± 0.15 ^gh^	146.45 ± 1.47 ^i^	155.65 ± 1.73 ^h^	196.92 ± 9.60 ^cd^	163.58 ± 0.39 ^g^	200.91 ± 8.34 ^c^	164.03 ± 3.90 ^g^	227.91 ± 0.89 ^b^	139.80 ± 0.09 ^j^
*Δ^7^*-Avenasterol	558.19 ± 3.12 ^a^	384.74 ± 0.23 ^k^	519.78 ± 0.75 ^b^	481.04 ± 1.20 ^f^	461.71 ± 0.84 ^g^	447.92 ± 1.35 ^h^	380.04 ± 3.90 ^l^	393.38 ± 0.72 ^j^	294.34 ± 4.68 ^n^	461.92 ± 4.04 ^g^	514.39 ± 0.68 ^c^	342.20 ± 0.13 ^m^	485.94 ± 1.85 ^e^	398.99 ± 0.84 ^i^	496.13 ± 2.83 ^d^	398.58 ± 1.18 ^i^

^1^ Values were means ± SD. Different letters in a line indicate significant differences (*p* < 0.05).

## Data Availability

The original contributions presented in the study are included in the article/Supplementary Material, further inquiries can be directed to the corresponding authors.

## Supplementary Materials

The following supporting information can be downloaded at: https://www.mdpi.com/article/10.3390/foods14122007/s1, Table S1: The phenotypical information of sixteen oat cultivars.
