# Supplementary material for: Comparative Study of Chemical Compositions and Antioxidant Capacities of Oils Obtained from Sixteen Oat Cultivars in China
Source: Foods. 2025 Jun 6;14(12):2007. doi: 10.3390/foods14122007 (PMC12192038; doi:10.3390/foods14122007)
Supplement: Supplementary file 1 [file foods-14-02007-s001.zip › foods-3641129-supplementary.pdf]

Supplementary table

Table S1 The phenotypical information of sixteen oat cultivars.

Name	Growth period duration (d)	Plant height (cm)	Ear length (cm)	1000-grain weight (g)	Feature
1#	85	103.2	13.4	14.2	lodging-resistant
2#	81	99.5	19	30	disease-resistant and drought
3#	76	80.6	15	23.7	stem hard and lodging-resistant
4#	83	107	19.8	27.5	lodging-resistant
5#	81	78.3	16.8	25.4	stem hard and lodging-resistant
8#	70	104	19	20.87	extremely early-maturing
9#	75	102.7	19	19.4	early-maturing
10#	75	101.4	18.1	20.7	early-maturing
11#	83	95.3	19.5	25.57	disease-resistant
13#	83	102	16.2	25.8	pests and disease-resistant
15#	73	91	15.5	21.8	pests and disease-resistant
16#	82	101.5	18.4	26.6	disease-resistant
18#	82	114.6	20.4	28.1	disease-resistant
20#	85	109.2	17.2	26.5	disease-resistant and drought
22#	85	108.7	15.8	22.2	strong tillering
24#	82	120.25	24	23.85	lodging-resistant
